# Conv3D-Based Video Violence Detection Network Using Optical Flow and RGB Data

**DOI:** 10.3390/s24020317

**Published:** 2024-01-05

**Authors:** Jae-Hyuk Park, Mohamed Mahmoud, Hyun-Soo Kang

**Affiliations:** 1 Department of Information and Communication Engineering, School of Electrical and Computer Engineering, Chungbuk National University, Cheongju-si 28644, Republic of Korea; 2022234002@cbnu.ac.kr (J.-H.P.); mohamedabokhalil@aun.edu.eg (M.M.); 2 Information Technology Department, Faculty of Computers and Information, Assiut University, Assiut 71515, Egypt

**Keywords:** deep learning, CCTV anomaly detection, optical flow, attention network

## Abstract

Detecting violent behavior in videos to ensure public safety and security poses a significant challenge. Precisely identifying and categorizing instances of violence in real-life closed-circuit television, which vary across specifications and locations, requires comprehensive understanding and processing of the sequential information embedded in these videos. This study aims to introduce a model that adeptly grasps the spatiotemporal context of videos within diverse settings and specifications of violent scenarios. We propose a method to accurately capture spatiotemporal features linked to violent behaviors using optical flow and RGB data. The approach leverages a Conv3D-based ResNet-3D model as the foundational network, capable of handling high-dimensional video data. The efficiency and accuracy of violence detection are enhanced by integrating an attention mechanism, which assigns greater weight to the most crucial frames within the RGB and optical-flow sequences during instances of violence. Our model was evaluated on the UBI-Fight, Hockey, Crowd, and Movie-Fights datasets; the proposed method outperformed existing state-of-the-art techniques, achieving area under the curve scores of 95.4, 98.1, 94.5, and 100.0 on the respective datasets. Moreover, this research not only has the potential to be applied in real-time surveillance systems but also promises to contribute to a broader spectrum of research in video analysis and understanding.

## 1. Introduction

Identifying violent conduct within video content has quickly become critical in public safety and security research. In the society of today, the widespread integration of closed-circuit television (CCTV) for crime prevention and surveillance has reached unprecedented levels. However, this expansion in CCTV deployment has not been matched by a corresponding increase in human resources for the monitoring and oversight of these systems. Consequently, automated systems that can rapidly and precisely detect instances of violence in real time are vital. These systems can provide a timely alert to hazardous scenarios, facilitating swift responses.

Traditional methods for detecting violence predominantly concentrate on manual feature extraction from video data [1,2,3]. However, these approaches frequently exhibit shortcomings in robustness and adaptability when deployed in real-world settings. This limitation stems from the intricate interplay of factors such as installation angles, diverse locations, varying backgrounds, and video resolutions of CCTV footage. In recent times, propelled by advances in artificial intelligence, several deep-learning models have been developed. These models autonomously identify features and patterns that were previously challenging to detect using conventional violence-detection methods [4,5,6,7].

Assessing the extent of motion plays a pivotal role in identifying violent occurrences in video content. Optical-flow techniques are commonly used to capture such motion patterns. This approach quantifies the movement between successive frames, proving effective in swiftly evolving scenes or instances of violence. However, optical-flow frames alone may not comprehensively detect violent scenarios. RGB frames offer indispensable cues to recognize violence within static images, revealing details about the elements in the scene and their interactions. For instance, a motion like running might be construed as benign, as what may be jogging in one context could signify a potentially hazardous situation in another. Herein lies the significance of RGB data. Analyzing color and object patterns within the frames enables a violence-detection model to comprehend a wider context, thereby minimizing false alarms. The insights obtained from the fusion of optical flow and RGB data are mutually complementary. Concurrently integrating both types of data can significantly bolster the efficiency and precision of violence detection by providing a more comprehensive understanding of the content.

For successful integration of optical flow and RGB data, a model that adeptly comprehends the interconnections between temporal and spatial dimensions becomes imperative. Convolutional neural networks (CNNs) have demonstrated their efficacy in handling spatial data in various applications, such as image recognition [8,9,10,11], alongside applications in security and image inpainting [12,13]. In the realm of video analysis, 3D CNN (Conv3D) models have gained prominence. These models consider the width, height, and temporal sequences (frames) of images, effectively handling both the temporal and spatial information inherent in videos. This concurrent processing of spatiotemporal aspects marks the capability of Conv3D models in video analysis and recognition.

This study introduces a robust violence-detection network tailored for diverse backgrounds. We focus on isolating and scrutinizing moving objects within videos rather than the static background, thereby enhancing detection capabilities across various datasets that portray violent scenarios. To capture features associated with non-background moving objects, we harness optical-flow features extracted from the video and complement these with RGB frames to comprehensively interpret the visual context. Both components serve as inputs to the model. Our methodology integrates these combined features to understand the spatiotemporal characteristics embedded within the video frames. To achieve this, we employ a Conv3D-based ResNet model, known as ResNet-3D, renowned for its proficiency in comprehending video data. This specialized model allows our system to be trained to recognize patterns of violence, considering both dynamic movements and visual context. By capitalizing on the strengths of optical flow and RGB data features, our approach represents a significant step towards developing a more accurate and reliable violence-detection model. This model surpasses existing models by addressing the challenges posed by the varying specifications of real-world CCTV setups, thereby offering enhanced robustness and adaptability.

The key contributions outlined in our study are as follows:

1. Extraction of motion information between frames using optical-flow features;
2. Understanding of visual contexts, such as objects and interactions, using RGB data;
3. Development of a model that captures the relationship between integrated RGB and optical-flow frames, and understands both spatial and temporal dimensions, using ResNet-3D and an attention module.

The remainder of the paper is structured as follows. Section 2 provides an in-depth exploration of crucial technologies fundamental to developing a violence-detection system. Section 3 outlines the algorithm designed to proficiently detect instances of violence. Section 4 emphasizes the presented data and preparatory steps for training, covering aspects such as loss functions and evaluation metrics. Section 5 evaluates the performance of the model using CCTV data. Finally, Section 6 concludes the paper, summarizing the research findings of this paper.

## 2. Related Work

In this section, we provide an overview of violence-detection techniques and focus on the preliminary studies for the method proposed in this paper. We discuss research on optical flow, studies combining optical flow with RGB data, Conv3D, and the trends in video-violence detection research.

### 2.1. Utilization of Optical Flow in Violence Detection

Recently, the importance of using optical flow, which extracts critical information about the dynamic movements of objects in violent situations, has been increasingly highlighted. Early research in violence detection applied to human action recognition [14] used traditional computer-vision techniques to extract optical flow by employing the dense trajectories method, which calculates trajectories of uniformly sampled points across frames to capture extensive optical flow information. Such traditional methods had limitations in precisely extracting optical flows from videos with various resolutions or containing numerous objects, resulting in violence-detection models with low performance in terms of accuracy and speed.

To overcome the limitations of these traditional computer-vision techniques, various deep-learning models that efficiently extract optical flows from image frames have been developed. There are three types of studies: studies that use spatial pyramid networks to estimate optical flows [15], studies that assume that most scenes in videos consist of rigid objects and estimate optical flow in such environments [16], and those that apply the transformer architecture for optical-flow estimation [17]. These deep-learning models have demonstrated high action-recognition rates, which have been widely acknowledged. The trend in violence-detection research is continuing towards enhancing the efficiency and accuracy of video analysis using optical flow.

### 2.2. Combining Optical Flow with RGB Data

Recent research has focused on combining optical flow with RGB data to gain an integrated understanding of the motion characteristics and visual context within video data [18]. Integrating features from RGB and optical flow allows for capturing various aspects and characteristics of video data, enabling the model to better understand the complex structures or patterns within the data. Moreover, it prevents the model from relying too heavily on specific information and allows it to learn more general patterns based on diverse information, thus reducing the risk of overfitting.

Several deep-learning models use both optical flow and RGB data. For instance, the I3D model [18], designed for video classification and action recognition, has shown high performance in video recognition using optical flow and RGB simultaneously as inputs. In the video object recognition approach proposed by Zhang [19], issues such as motion blur, defocus, partial occlusion, and rare poses in videos were addressed by combining optical flow with RGB data. Studies that combined RNN [20] and CNN models to learn temporal features [19] also used both optical flow and RGB data. Additionally, various video-classification models have used RGB and optical flow [21,22,23].

### 2.3. Conv3D

Conv3D is gaining significant attention in the field of video processing, particularly in action recognition within videos. Unlike traditional CNNs, which focus on 2D data such as images, Conv3D is designed to detect both the static content of images and the dynamic changes over time. The key to this capability is that Conv3D performs convolutions over time and across width and height, which is typical in CNNs. Owing to this feature, Conv3D can simultaneously capture and learn the temporal continuity and spatial characteristics of videos.

Consequently, the amount of research that uses Conv3D for video data analysis is increasing. The TSN model [24], a new approach that more precisely represents motion within videos, is an example. TSN segments full videos into multiple parts and integrates the sampled frame information from each segment to derive a comprehensive representation of the videos. This approach has improved the accuracy of human-action recognition in videos. Christoph [25] introduced a ResNet-based Conv3D architecture, similar to the one used in this study, to explore methods of processing spatiotemporal information in videos. Early Conv3D [26] application studies presented research that improved the way of learning features through convolutions in the spatiotemporal dimensions of videos. Another study [27] proposed the C3D model, exploring a methodology for directly learning spatiotemporal features from entire video clips. This model deeply analyzed the relationships and patterns between frames using continuous convolutions in the temporal dimension.

Thus, research centered around Conv3D is achieving high performance in action recognition while overcoming the spatiotemporal complexity of videos. This significantly expands its potential applications in various fields related to video processing.

### 2.4. Video-Violence Detection

Automatic detection of violent behaviors in videos has become important in various sectors, such as security, education, and transportation. In the early stages of video-violence detection research, features extracted manually from the videos were used to develop detection models. One of the seminal works proposed by Hansner [4] used handcrafted features to detect violent actions in crowds in real time. It is a representative example of early violence-detection models. Although these techniques achieved considerable recognition rates with simple features in specific video environments, they fell short in detecting violent patterns in the complex settings of various CCTV systems.

With advances in technologies such as deep learning, end-to-end models that extract features directly from video data are being proposed. These methods, with the significant increase in the amount of data available for model training, have shown much higher performance than traditional handcrafted-feature-based approaches and are highly accurate and reliable in real-world environments.

Various deep-learning-based violence-detection models, such as the Conv-AE [6] model, learn temporal consistency in video sequences by combining the feature extraction capability of CNNs with the data reconstruction ability of AutoEncoders [5] after compressing the data. Another example is Explainable VAD [7], a violence-detection network based on unsupervised learning that learns general knowledge from video data and detects abnormal events in specific contexts. Moreover, the skeleton-based approach [28] abstracts actions using human skeletons to form continuous trajectories in each frame and uses this information to detect anomalies in movement.

Research on video-violence detection has evolved from early handcrafted-feature-based methods to now leveraging deep learning to utilize various features comprehensively. This technological progression plays a crucial role in accurately detecting acts of violence in more diverse and complex environments.

## 3. Approach

**Figure 1:**
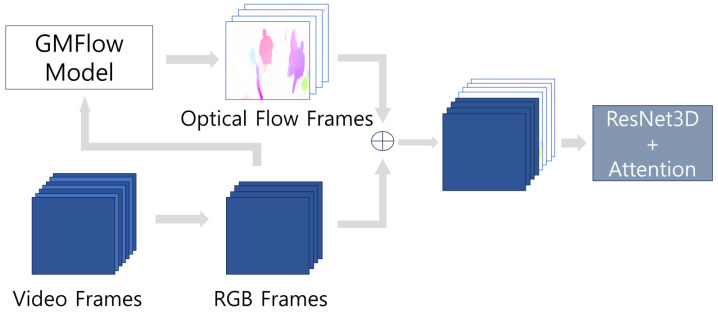
Proposed violence detection network.

We propose a violence-detection model that combines RGB and optical-flow data using a Conv3D model based on ResNet. The structure of the proposed violence-detection model is shown in Figure 1. Given video data, RGB frames are extracted at regular frame intervals. Subsequently, optical-flow frames are derived from these RGB frames using a deep-learning model; then, the RGB and optical-flow frames are combined. The combined frames are fed into the backbone model, Conv3D + attention module, for violence-detection classification. The specific details of the proposed model are described below.

### 3.1. Optical Flow

Optical flow represents the pixel movement between two consecutive image frames as a vector field and is crucial for detecting the movement of objects or the background, as well as camera motion. Violent actions are characterized by abrupt and unpredictable movements, which can be analyzed using optical flow. Understanding various patterns of movement during violent interactions through optical flow enables more accurate detection of the intensity and nature of the behavior. The precision and efficiency of the model in violence detection can be enhanced using the pattern changes in optical flow corresponding to violent behaviors in different environments.

**Figure 2:**
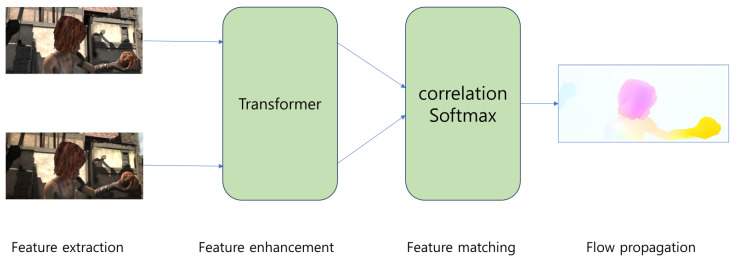
GMFlow model used for optical flow extraction.

**Figure 3:**
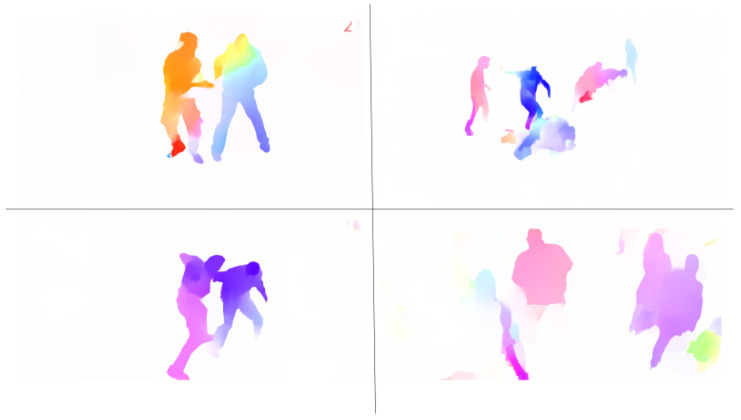
Optical-flow image extracted from a violent situation video.

We employed GMFlow [29], a state-of-the-art model that achieved the highest accuracy on the datasets used for validating optical-flow model performance, such as Sintel [30] and KITTI [31]. GMFlow uses a pre-trained model with the Sintel dataset, which consists of videos with various backgrounds and their corresponding optical flow. The model structure of GMFlow is shown in Figure 2. Figure 3 illustrates the resulting image of optical flow obtained from a video of a violent situation, emphasizing the dynamic changes between video frames. These highlight regions of movement, mainly detecting features of moving objects. Optical flow visually represents these patterns and directions of movement, aiding in a clearer understanding of changes in moving objects or scenes.

### 3.2. RGB Data

In addition to optical flow, which captures dynamic movements in videos, RGB data should also be used to capture static context information effectively. RGB data contain the original color information of the video, which can identify static information such as the background, objects, and environment of violent behavior. Since violent situations are often closely linked to specific backgrounds or contexts, this static information provides critical insight for increasing the detection accuracy. For example, the act of one person pushing another could have a significantly different contextual meaning when occurring in a park, suggesting play, as opposed to in a parking lot, suggesting violence. Thus, RGB data provide the context of the action, helping to ascertain the intent or cause of action more accurately.

### 3.3. Conv3D

Conv3D is a convolutional operation designed for 3D data, especially temporal data such as videos. While traditional CNNs only perform convolutions across the spatial dimensions of height and width of an image, Conv3D additionally considers the temporal dimension, performing convolutions across three dimensions: height, width, and time.

**Figure 4:**
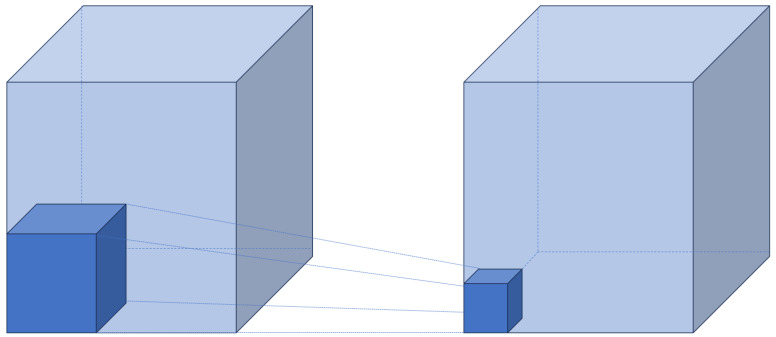
Conv3D calculation.

Figure 4 visualizes the operation of Conv3D. The process of Conv3D operation proceeds as follows:

1. The input is a 4D tensor of the form C × T × W × H, where C represents the number of channels (3), T represents the temporal dimension (frames), H represents the height of the image, and W represents the width of the image.
2. For the convolution filter, a 5D tensor of the form F × C × T × H is used. Here, F is the number of filters. The depth of the filter must match the number of input channels.
3. When performing the convolution, a 3D filter is applied to each channel of the input data to generate output values. The filter slides along the temporal dimension, computing spatial convolutions at each position. These results are summed to obtain the final output value.
4. The output is a 4D tensor of the form F × T × W × H, composed of the convolution results for each filter.

Through these 3D operations, Conv3D can extract not only the features of images but also the information between frames along the time axis.

### 3.4. ResNet-3D

ResNet-3D [25] is an architecture that extends the core principles of the existing CNN model, ResNet, and applies it to the Conv3D model. This allows the model to capture temporal continuity between video frames. Such a feature enables the model to effectively learn patterns and information in the temporal dimension.

The essential feature of ResNet, the residual connection, is also applied to this ResNet-3D structure. One of the main advantages of residual connections is that they alleviate the vanishing gradient problem, which is common in deep networks. This issue can become even more pronounced in complex networks that handle high-dimensional data, such as videos in the case of Conv3D. With the introduction of residual connections, stable improvements in model performance are possible even in deep Conv3D architectures.

**Figure 5:**
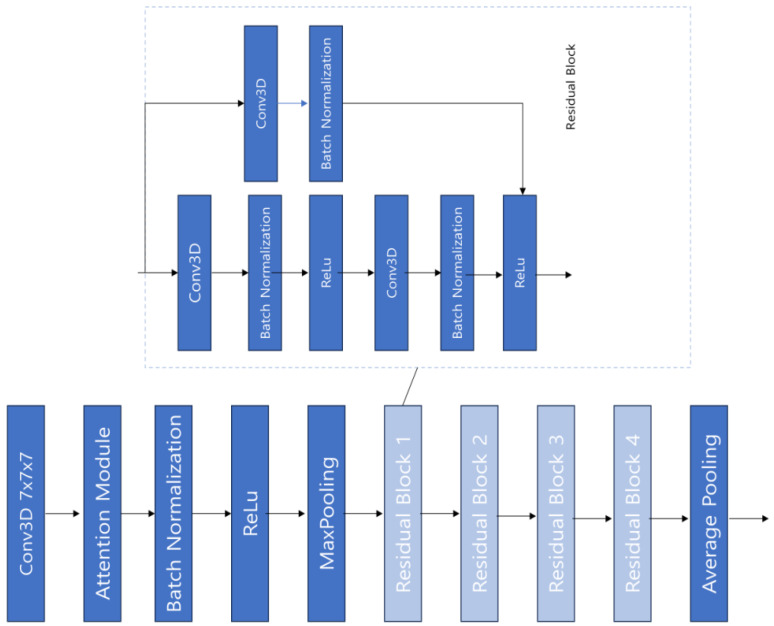
ResNet-3D model structure.

Because ResNet-3D processes information in the temporal dimension, it can intricately learn information about dynamic characteristics such as movement, continuity, and temporal changes, demonstrating superior performance on video analysis problems like action recognition compared with other Conv3D models. Figure 5 below depicts the structure of the ResNet-3D model used in this study.

As shown in Figure 5, ResNet-3D uses residual connections to mitigate the vanishing gradient problem by adding skip connections that directly add the input to the output of each layer, aiding the network in learning more effectively. It also employs bottleneck structures at intervals to reduce the computational load and uses batch normalization to enhance network stability. We used ResNet-3D based on Conv3D as the backbone model, focusing on learning the temporal continuity of video data to extract more sophisticated features.

### 3.5. Attention Module

**Figure 6:**
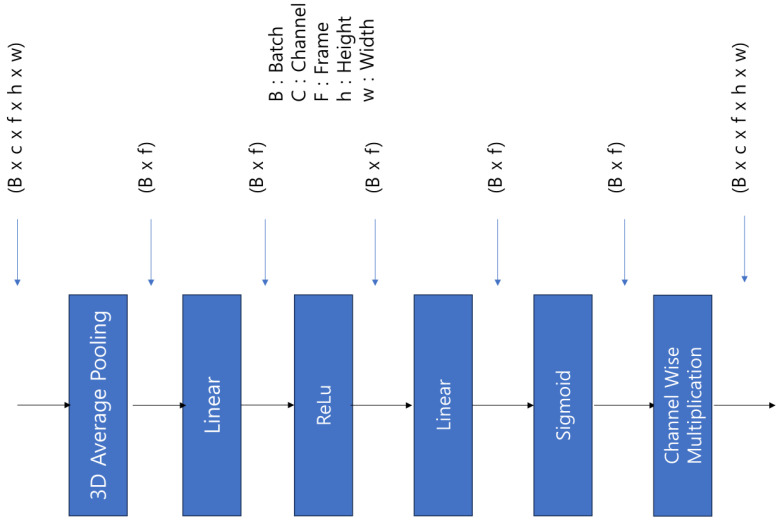
Attention module structure.

To detect violent situations, it is crucial to capture the dynamic changes between consecutive frames within the video and the significant frames related to dangerous visual information. We used data in the form of a combination of RGB frames and optical flow and employed the SEModule3D attention module within our backbone model, ResNet-3D, to assist the model in identifying important frames from the combined data. SEModule3D is an adaptation of the widely used squeeze-and-excitation (SE) attention module in images for 3D data, modeled to suit 3D information. The SE network was first proposed in [32], and its basic concept is to explicitly model the interdependencies between channels of feature maps. This allows the network to learn the importance of each Frame, thereby enhancing the representativeness of the network. The operation of SEModule3D is shown in Figure 6.

The operational structure of SEModule3D is as follows:

1. Squeeze: It uses average pooling to extract the global average information of each Frame.
2. Excitation: Based on the global average statistics, the feature map is transformed through two fully connected layers. The first layer reduces the number of Frames, followed by an adjustment to values between 0 and 1 via the rectified linear unit and the sigmoid functions.
3. Rescale: The final output is obtained by multiplying the input feature map with the sigmoid output. This process enables the network to learn the importance of each Frame, emphasizing important Frames and suppressing less important ones.

The attention module helps the violence-detection model to focus on important Frames within the input data, effectively capturing critical areas within specific frames and important movements between consecutive frames.

## 4. Experiment

In this section, we introduce the experimental datasets, describe the loss functions and optimization techniques employed, and provide an overview of the actual experimental setup.

### 4.1. Training Data

To train the model to detect violent behaviors, we used training data that include a variety of backgrounds and situations captured by real CCTV footage. Each dataset was divided into an 8:2 ratio for training and validation purposes. The datasets used are as follows:

UBI-FIGHT [33]: This contained real violent situations occurring in various places and consisted of 1000 data samples. Among these, 786 were normal situations and 214 were abnormal, making it an imbalanced dataset.

Hockey [34]: This contained violent situations from real hockey games, comprising 1000 data samples with 500 normal and 500 abnormal situations.

Movie-Fights [35]: This contained 200 violent situations captured in various backgrounds from movies—100 normal and 100 abnormal.

Crowd [4]: A video dataset about collective violence situations. It contained 256 data samples, with 123 normal and 123 abnormal situations.

### 4.2. Loss Function

For training, a loss function is used as a metric to gauge the prediction and error. A loss function refers to a function that calculates the error based on the difference between input and output values. Owing to the nature of the data used in anomaly detection, which often involves an imbalance between normal and abnormal data, the focal loss function [36] is employed to address this imbalance as much as possible. It aids the handling of imbalanced class distributions in binary and multi-class classification problems and performs effectively for datasets with imbalanced distributions, which are not adequately addressed by standard cross-entropy loss.

Focal loss is based on cross-entropy loss; however, it assigns greater weight to misclassified classes and reduces the loss for correctly classified classes. This encourages the model to focus on classes that are difficult to classify and less learned. Below is the focal loss formula.

(1) $$FL\left(p_{t}\right)=-\alpha_{t}{\left(1-p_{t}\right)}^{\gamma }\log(p_{t})$$

The essence of focal loss is as follows. During the model training, if a sample is deemed easy, the value of $p_{t}$ in the formula becomes high, and by adding ${\left(1-p_{t}\right)}^{\gamma }$ to the loss, a penalty is imposed for high-confidence predictions. Here, γ is known as the focusing parameter, and it serves to reduce the contribution of easy samples to the loss during model training.

### 4.3. Optimization Technique

We employed AdamW [37], an improved version of adaptive moment estimation (Adam) [38], to address the issue of overfitting caused by training data from imbalanced datasets.

#### 4.3.1. Adam

Adam is one of the deep-learning optimization algorithms, which combines the advantages of momentum [39] and RMSprop [40]. Adam adjusts the learning rate for each parameter using the average of the past squared gradients.

Adam is an optimization method that adaptively adjusts the learning rate for each parameter, following the update rule:

(2) $$\theta_{t+1}=\theta_{t}-\frac{\eta }{\sqrt{{\hat{v}}_{t}}+\epsilon }{\hat{m}}_{t},$$

- θ represents the parameters to be optimized;
- *t* is the current time step;
- η is the learning rate;
- ${\hat{v}}_{t}$ and ${\hat{m}}_{t}$ are bias-corrected estimates of the first and second moments of the gradients, respectively;
- ϵ is a small scalar added to improve numerical stability.

The bias-corrected estimates, ${\hat{m}}_{t}$ and ${\hat{v}}_{t}$, are computed as follows:

(3) $$\begin{array}{cc} {\hat{m}}_{t} & =\frac{m_{t}}{1-\beta_{1}^{t}}, \end{array}$$

(4) $$\begin{array}{cc} {\hat{v}}_{t} & =\frac{v_{t}}{1-\beta_{2}^{t}}, \end{array}$$

where $m_{t}$ and $v_{t}$ are the first moments (the mean) and the second moment (the uncentered variance) of the gradients, and $\beta_{1}$ and $\beta_{2}$ are the decay rates for these moments.

#### 4.3.2. AdamW

AdamW is a modification of the Adam update rule, incorporating L2 weight decay [41]. L2 weight decay is a regularization method that limits the complexity of the model to prevent overfitting. The update rule of AdamW is similar to that of Adam, but includes an additional weight decay term during the parameter update phase. The formula for AdamW is as follows:

(5) $$\theta_{t+1}=\theta_{t}-\frac{\eta }{\sqrt{{\hat{v}}_{t}}+\epsilon }\left({\hat{m}}_{t}+\lambda \theta_{t}\right)$$

- θ denotes the parameters of the model;
- *t* indexes the current time step;
- η is the learning rate;
- ${\hat{m}}_{t}$ is the bias-corrected estimate of the first moment (the mean) of the gradients;
- ${\hat{v}}_{t}$ is the bias-corrected estimate of the second moment (the uncentered variance) of the gradients;
- ϵ is a small scalar used to prevent division by zero and ensure numerical stability;
- λ represents the weight decay coefficient.

Here, $\lambda \theta_{t}$ is the parameter for L2 weight decay, which prevents overfitting by subtracting a small value from the weight updates. Datasets like those for violence detection often consist of imbalanced data, where the number of normal instances far exceeds that of abnormal ones. Therefore, to mitigate the problem of overfitting due to training on imbalanced datasets, we employed the AdamW optimization technique.

#### 4.3.3. Training Environment

The training was conducted on Windows 11 using an i7-13700K CPU, an NVIDIA GeForce RTX 4090 graphics card, and Pytorch 2.00 + CUDA 11.7 version.

#### 4.3.4. Evaluation Metrics

Accuracy and the receiver operating characteristic–area under the curve (ROC-AUC) [42] were used as metrics to evaluate the performance of the model. Accuracy is one of the most intuitively used performance evaluation metrics in classification problems. It is calculated as the ratio of correct predictions to the total number of predictions. Below is the formula for accuracy.

(6) $$\mathrm{Accuracy}=\frac{\mathrm{TP}+\mathrm{TN}}{\mathrm{TP}+\mathrm{TN}+\mathrm{FP}+\mathrm{FN}}$$

Here, True positive (TP) is when the actual class is positive and the model also predicts positive. True negative (TN) is when the actual class is negative and the model predicts negative. False positive (FP) is when the actual class is negative but the model predicts positive. False negative (FN) is when the actual class is positive but the model predicts negative.

The ROC curve graphically represents the relationship between sensitivity and specificity. It allows the performance of a model to be evaluated across various classification thresholds. Below are the formulas for sensitivity and specificity.

(7) $$\mathrm{Sensitivity}\;\left(\mathrm{TPR}\right)=\frac{\mathrm{TP}}{\mathrm{TP}+\mathrm{FN}}$$

(8) $$\mathrm{Specificity}\;\left(\mathrm{TNR}\right)=\frac{\mathrm{TN}}{\mathrm{TN}+\mathrm{FP}}$$

The AUC represents the area under the ROC curve. The closer the AUC value is to 1, the better the model performance. If the AUC value is close to 0.5, the performance of the model is considered to be at the level of random guessing.

## 5. Experimental Result

We used accuracy and AUC as the evaluation metrics for performance comparison between our model and the models in [6,18,29,43,44,45,46] that have shown excellent performance in various violent-situation detection in recent studies. In datasets like Hockey, Movie-Fights, and Crowd, where the ratio of abnormal to normal data is evenly distributed, model performance was evaluated based on accuracy (ACC).

On the other hand, in the case of datasets with a large imbalance between normal and abnormal data, such as UBI-FIGHT, it is difficult to evaluate model performance using only conventional ACC. Since models can be biased towards the predominant class in imbalanced data, we adopted the AUC evaluation metric, which is widely used to assess performance on imbalanced datasets.

Deep networks, capable of extracting features across various layers, have demonstrated powerful performance on high-dimensional data like videos, but issues such as overfitting can occur, and depth does not always correlate with performance. We experimented with various depths of the backbone model, ResNet-3D, and conducted performance evaluations. Four versions of the widely used classification model proposed (ResNet-10, ResNet-34), were applied in the experiments. By using models of various depths, we sought to explore the relationship between the depth of each model and its performance.

The results in Table 1 are from an experiment using the UBI-FIGHT dataset. For evaluation, the model proposed by Hasan [6], which achieved high performance in the field of violent-situation detection; the model proposed by Ravanbakhsh [43]; Wang’s model [44]; the Binary SVM Classifier proposed by Sultani [45]; and the GMM model [47], which exhibits excellent performance in video violence-situation recognition, were compared. Before training and evaluating, the data were resized to 512 × 512, which is a power of two, ensuring efficient operation in GPU memory allocation and thread management. Since the number of frames in each video within the video dataset can vary, 150 frames were extracted from the frames of each video to train the model. The UBI-FIGHT dataset comprised 784 normal videos and 214 abnormal videos and included a vast array of video lengths ranging from a minimum of one minute to a maximum of one hour. However, due to the significant imbalance between the number of normal and abnormal videos, the AUC metric, which is more suitable for imbalanced datasets, was used instead of the commonly used accuracy (ACC) metric to evaluate the performance of the model. The model based on the proposed ResNet-34 showed outstanding performance, with an AUC score of 0.952.

Table 2 presents the experimental results for the Hockey, Movie-Fight, and Crowd datasets. The models used for comparison, which are I3D, Flow Gated, and SPIL, are all networks that have shown good performance in violence-situation detection. Since all the data used in Table 2 are balanced datasets with equal proportions of normal to abnormal instances, accuracy (ACC) was used as the evaluation metric for analysis. In the Hockey dataset, the model proposed in this paper, Proposed (ResNet-34), achieved high performance with an ACC of 98.1. In the Movie-Fights dataset, all depths of ResNet models reached high performance with an ACC of 100. For the Crowd dataset, which involves violent situations occurring within large crowds, the Proposed (ResNet-10) model showed good results with an ACC of 94.0, close to the performance of SPIL.

Table 3 presents a comparative analysis of performance with and without the use of an attention module. The results indicate a higher ACC when the attention module is employed. It demonstrates that assigning weights to significant frames among RGB and optical-flow frames enhances the effectiveness of video violence detection. This finding confirms the value of focusing on crucial frames in improving the recognition of violent situations in videos.

Additionally, in terms of time efficiency, the proposed model demonstrated reliable performance with a processing speed of 30–60 frames per second (FPS), aligning well with the operational standards of real-time CCTV systems. Although this aspect was not directly compared with other models, it adds a significant dimension to the practical applicability of our approach in real-world scenarios.

## 6. Conclusions and Future Work

In this study, we propose an enhanced violence-detection model that integrates optical flow and RGB data by augmenting the ResNet-3D model with an attention module. This model effectively captures both the spatiotemporal features of the dynamic context from optical flow and the spatial context of RGB frames, combining them for a comprehensive analysis. The introduction of the attention module significantly improved the model’s ability to focus on crucial frames for detecting violent situations within the combined RGB and optical-flow frames, thus increasing the accuracy of violence detection.

While the foundational ResNet-based ResNet-3D model, which leverages the residual connections of ResNet, allows for efficient training with high-dimensional data such as videos. Performance evaluations on real-life CCTV datasets, including the Crowd, Hockey, UBI-FIGHT, and Movie-Fights datasets, have shown the superior accuracy of our model over existing models in various environments with differing backgrounds, lighting, and movements. However, the model’s performance slightly decreases in scenarios involving videos with a high density of people. Additionally, while the model is capable of processing video in real-time, reducing the memory required to compute optical flow from images remains a challenge for us.

Our future research plans include two primary directions. Firstly, we aim to conduct research on reducing memory usage for optical flow extraction. This is a crucial step to enhance the efficiency of video processing and increase the applicability of real-time systems. Specifically, by improving memory efficiency, we intend to make optical-flow-based models more versatile across a wider range of equipment and environments.

Secondly, we plan to focus on increasing the accuracy of violence detection in environments with spatial constraints, like crowded areas. Such settings pose a unique challenge to violence detection models due to the difficulty in detecting dynamic movements. To address this, we will work on developing more sophisticated data processing techniques and algorithms to optimize the model for high accuracy in complex environments.

Our findings demonstrate that by utilizing optical flow and the attention module, the model is able to focus more on the dynamic aspects of objects, showing robust performance even in video datasets with diverse backgrounds. This video violence detection model is expected to play a significant role in real-time security and surveillance systems in the future, especially in enhancing safety in complex urban environments or large public spaces. Furthermore, the development of this model expands the possibilities of AI-based security systems and paves the way for the development of more sophisticated surveillance technologies. This represents a significant advancement in improving public safety, offering a swift and efficient mechanism for responding to hazardous situations.

## Figures and Tables

**Table 1:** Performance comparison of proposed model and existing models on the UBI-FIGHT dataset.

Method	AUC
Hasan et al. [6]	0.510
Ravanbakhsh et al. [43]	0.523
Wang et al. [44]	0.610
Sultani et al. [45]	0.892
GMM [47]	0.906
Proposed (ResNet-10)	0.951
Proposed (ResNet-34)	0.952

**Table 2:** Performance comparison of proposed model and existing models on the Hockey, Movie-Fights, and Crowd datasets.

Method	Hockey (ACC)	Movie-Fights (ACC)	Crowd (ACC)
I3D [6]	0.934	0.958	0.834
Flow Gated	0.980	0.973	0.888
SPIL [45]	0.968	0.953	0.954
Proposed (ResNet-10)	0.970	1.0	0.940
Proposed (ResNet-34)	0.981	1.0	0.915

**Table 3:** Comparative performance evaluation of models with and without attention module.

Method	Hockey (ACC)
Proposed (ResNet-10)	0.970
Proposed (ResNet-10) Without Attention	0.930
Proposed (ResNet-34)	0.981
Proposed (ResNet-34) Without Attention	0.925

## Data Availability

Data are contained within the article.
